# MEN1 Syndrome and Hibernoma: An Uncommonly Recognised Association?

**DOI:** 10.1155/2014/804580

**Published:** 2014-09-22

**Authors:** Venus Hedayati, Khin Thway, J. Meirion Thomas, Eleanor Moskovic

**Affiliations:** Royal Marsden Hospital, Fulham Road, London SW3 6JJ, UK

## Abstract

MEN1 syndrome is known to classically result in parathyroid, pituitary, and pancreatic islet cell tumours. However, the potential association of MEN1 syndrome with hibernoma, a benign tumour with differentiation towards brown fat, is far less well known, despite their genetic profile both being linked to deletion of the *MEN1* gene. Herein, we describe a case with its key radiological and pathological findings.

## 1. Case Presentation

**Figure 1:**
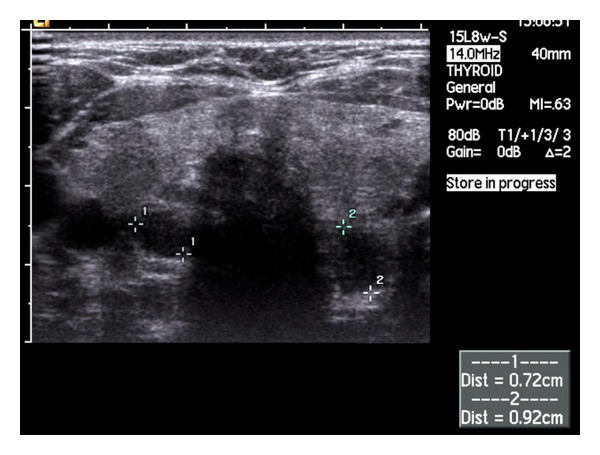
An ultrasound scan demonstrating hypoechoic nodules (outlined by caliper markings) inferior to both thyroid nodules highly suggestive of parathyroid adenomas.

A 40-year-old female first presented to an endocrinologist following a diagnosis of hypercalcaemia secondary to hyperparathyroidism. An ultrasound of the neck demonstrated 2 parathyroid adenomas (Figure 1) and a sestamibi scan confirmed a right inferior parathyroid tumour. Following a four-gland parathyroidectomy, chief-cell hyperplasia was demonstrated in all four glands.

**Figure 2:**
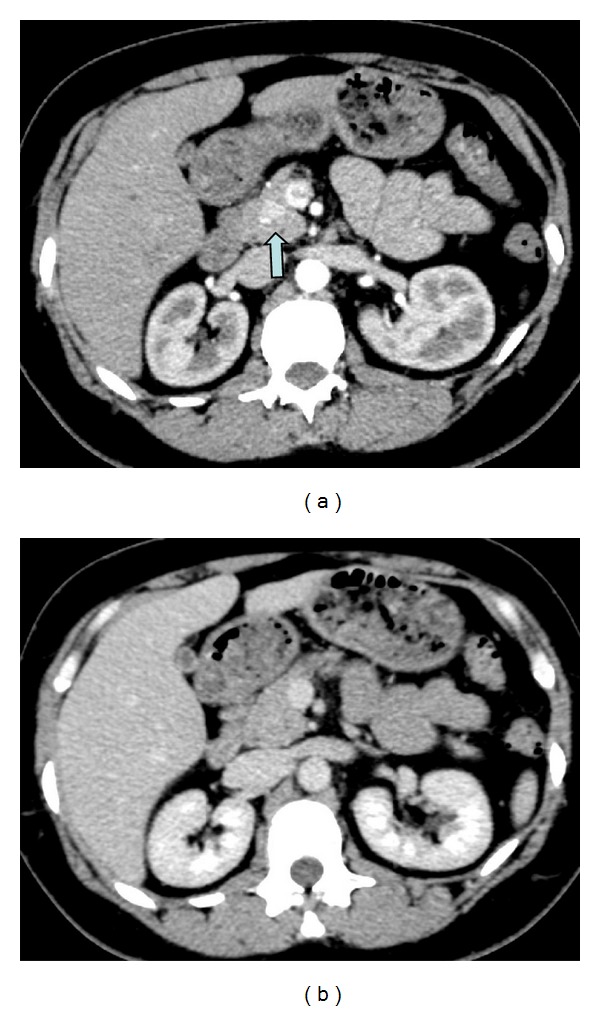
(a) Arterial phase scan through the abdomen demonstrates an enhancing lesion in the pancreatic head (arrow). (b) Venous phase: the lesion has become isoattenuating. Appearances are in keeping with a neuroendocrine tumour.

Within a year, the patient represented with episodes of dizziness, disorientation, impaired cognitive function, and, at times, irrational behaviour with pallor and perspiration. An MRI of the brain did not demonstrate any abnormality. However, a fasting proinsulin level was elevated above 49 (<11 pmol/L) and a fasting blood glucose dropped to 2.28 mmol/L with a normal C-peptide of 2.66 mcg/L raising the suspicion of an insulinoma. This was confirmed on a CT study of the chest, abdomen, and pelvis, which demonstrated a 14 mm arterialising lesion in the pancreatic head (Figure 2). Genetic testing revealed a pathogenic mutation in the* MEN1* gene, c1328C>A;p(Ser443Tyr).

**Figure 3:**
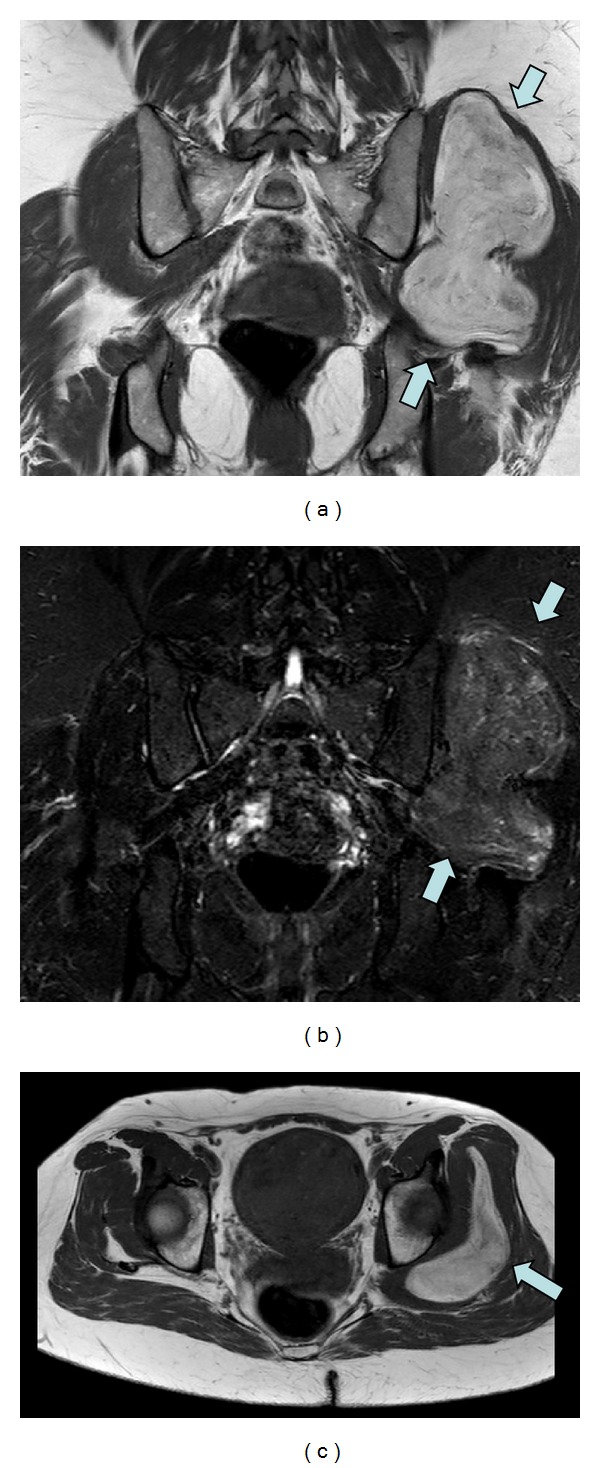
(a) Coronal T1, (b) coronal STIR, and (c) axial T1 MRI images demonstrate a lobulated mass of complex fat attenuation arising from the left gluteus medius muscle, outlined by blue arrows.

**Figure 4:**
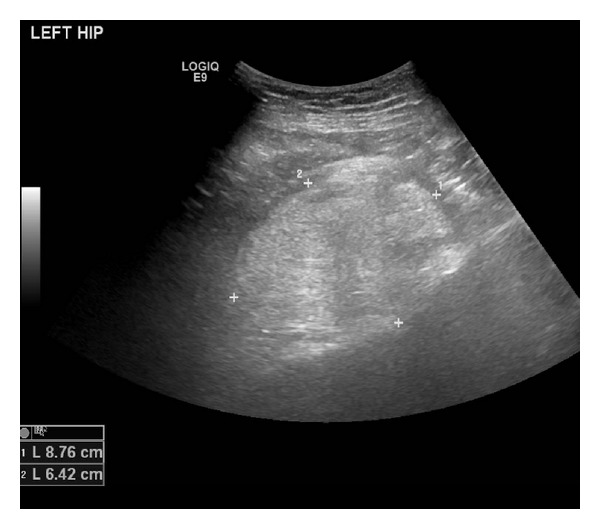
An ultrasound scan demonstrates a hyperechoic lesion, outlined by calliper markings. The homogenous echoic appearance is that of a fat containing lesion.

The CT study also demonstrated an enhancing mass in the left gluteal region. On further questioning, the patient's primary concern was of pain relating to the left hip, which was intermittent in severity and frequency but had increased over the preceding months. MRI demonstrated a 13 × 11 × 7 cm lobulated mass of complex fat signal arising from the left gluteus medius muscle (Figures 3 and 4). Internal vascularity and erosion of the underlying iliac blade raised the suspicion of an aggressive tumour.

**Figure 5:**
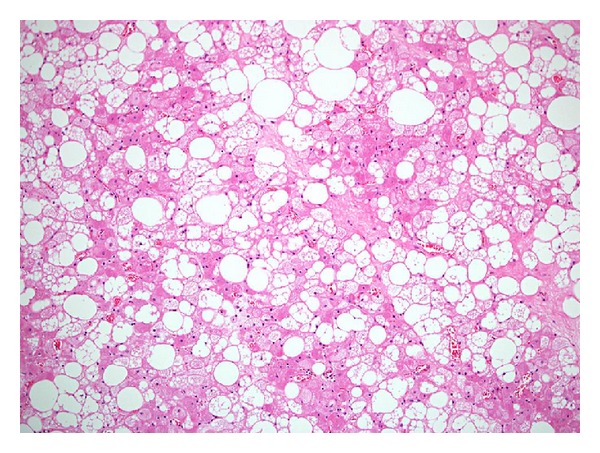
Hibernoma. Photomicrograph of the excision specimen shows a hibernoma with typical features of a prominent mixture of adipocytes of fetal type. There are only a few scattered large mature univacuolated adipocytes, but numerous multivacuolated adipocytes and smaller polygonal cells with abundant cytoplasm containing finely granular eosinophilic cytoplasm or numerous lipid vacuoles. Haematoxylin and eosin, ×100.

Ultrasound guided core biopsies of the left hip mass were taken to exclude a liposarcoma or complex haemangioma. The histology showed a neoplasm composed of sheets of brown fat cells, in keeping with the classical features of a typical subtype hibernoma (Figure 5).

## 2. Discussion

Hibernomas are rare benign tumours with differentiation towards brown fat. Their aetiology remains unknown and many occur at sites of normal brown fat in fetuses and newborns. The most common sites of residual fetal brown fat are in the mediastinum, posterior triangle of the neck, and periscapular regions, as often seen on FDG PET-CT. Similarly, hibernomas tend to occur in the shoulder, neck, chest wall, thighs, abdomen, and retroperitoneum. However they have also been identified at sites where brown fat is not known to preexist [1, 2].

The clinical presentation is of a slowly growing painful mass that may be warm on palpation due to high vascularity. They may locally invade, including striated muscle, or compress adjacent structures. However, by definition they are benign and neither metastasize nor recur once excised. This renders surgical excision treatment of choice in painful lesions. Age of presentation is most commonly between the 3rd and 4th decade and they are slightly more common in males.

Pure hibernomas, containing brown fat only, are less common than those comprising a combination of white and brown fat. Macroscopically they tend to be well-circumscribed, encapsulated, yellow-brown lobulated tumours, which can be as large as 30 cm. The histological composition varies depending on the ratio of univacuolated adipocytes of mature white fat and multivacuolated adipocytes of brown fat. There are four histological subtypes [3, 4] as described below, with percentage incidence in brackets, and many are S100 protein positive on immunohistochemistry [5]:

1. typical (nonlipoma type), with more than 70% multivacuolated adipocytes, which is the most common form of hibernoma (82%) that occurs most frequently in the thigh,
2. lipoma-like (7%), with <70% multivacuolate adipocytes, also more commonly found in the thigh,
3. myxoid (9%), containing a loose basophilic matrix, most common in the head and neck,
4. spindle cell (2%), with features of spindle cell lipoma, most frequent in the posterior neck and scalp.

Given the spectrum of histological features, it is of little surprise that the imaging characteristics are so variable, and on the basis of imaging alone they can be confused with more aggressive liposarcomas; several case reports of hibernomas mimicking liposarcomas have been reported [6]. The radiological differential diagnosis of both benign and malignant fat containing tumours, including atypical lipoma, haemangioma, angiolipoma, and liposarcoma, therefore warrants histological characterisation prior to excision.

On MRI they can be iso- or hypo-intense to fat on T1, iso- to hyper-intense to fat on T2 and iso- to hyper-intense to muscle on STIR. They often contain low signal internal strands and may be more heterogeneous on MRI depending on their histological composition [2, 7, 8]. Enhancement is common and more avid depending on the percentage of brown fat contained with the lesion. FDG-PET demonstrates highly metabolically active lesions.

MEN1 syndrome is usually an autosomal dominant inherited disorder that arises from germline mutation of the* MEN1 *gene (chromosome 11q13), which encodes menin, a protein required for transcriptional regulation and genomic stability. It is widely believed that* MEN1 *is a tumour suppressor gene, given that loss of the gene results in formation of tumours as in MEN1 syndrome. Despite usually being familial, there are sporadic mutations resulting in de novo MEN1 syndrome. The clinical syndrome is characterized by the development of parathyroid and pituitary adenomas and pancreatic endocrine tumours, amongst other manifestations. It is also thought that lipomatous tumours are more commonly represented in this population [9] and present in up to 30% of patients [10, 11].

Hibernomas are associated with rearrangements of chromosome bands 11q13-21 [12]. Translocation of 11q13 is thought to be the karyotypic profile of a hibernoma [13]. Nord et al. have shown that hibernomas display translocations involving 11q13 as well as deletion and transcriptional downregulation of the* MEN1* and* AIP* genes, both on 11q13; the latter gene is involved in familial pituitary adenoma syndrome [13]. Homozygous loss of* MEN1 *and, less commonly,* AIP* was found in the hibernomas they investigated. It is postulated that* AIP* suppresses peroxisome proliferator-activated receptor- (PPAR-) *α* and menin interacts with PPAR-*γ* which is found in brown and white adipocytes. The elimination of both menin and PPAR-*α* may lead to loss of regulation on brown fat production [3, 13]. It is already thought that loss of PPAR-*γ* function may lead to the overproduction of lipomas in patients with MEN1 syndrome [14].

It has been argued by Gisselsson et al. [15] that whilst aberrations of 11q13 have been demonstrated in hibernomas, tumorigenesis is through separate routes. The first reason they gave for this is that hibernomas are not known to occur in MEN1 syndrome, although lipomas and other mesenchymal tumours are. In addition, they stated that the endocrine tumours in MEN1 syndrome show more complex karyotypes which rarely involve chromosome 11 aberrations. Characteristic genetic changes involving 11q13 have not been found to be associated with lipomas or liposarcomas.

This case illustrates that hibernomas can be identified in patients with MEN1 syndrome. The incidence of hibernomas in patients with MEN1 syndrome has not been fully established; this might be because these are benign and slowly growing and therefore are less well documented. Recognition of this benign entity, particularly in this subset of genetically predisposed carriers, is important for the specialist clinician and radiologist, because of its ability to locally invade and also to mimic liposarcoma.

## 3. Key Points

- Hibernomas are rare, benign tumours with differentiation towards brown fat.
- They are associated with deletions in* MEN1* and* AIP* genes and therefore an association with MEN1 syndrome is postulated.
- Their radiological features can vary according to histological subtype, and they can mimic more aggressive tumours including liposarcoma.
- Recognition of their existence and relationship with MEN1 can alert the clinician and radiologist to this as an important benign differential diagnosis.

## References

[1] Gangarossa G, Sabato M, Pacciardi F A case of hibernoma Eurorad.

[2] Aniq H, Chakraborty S, Ritchie DA Hibernoma. EURORAD. Radiology Case Database.

[3] Grayson JW, Wallace JC (2012). A case report and discussion of hubernomas: pathology, genetics, diagnosis and treatment. *American Journal of Clinical Medicine*.

[4] Balaguera J, Fernandez I, Aquiriano L (2010). Axillary Hibernoma : an usual beningn sift tissue tumour. *The Internet Journal of Surgery*.

[5] Moretti VM, Brooks JSJ, Lackman RD (2010). Spindle-cell hibernoma: a clinicopathologic comparison of this new variant. *Orthopedics*.

[6] Gaskin CM, Helms CA (2004). Lipomas, lipoma variants, and well-differentiated liposarcomas (atypical lipomas): results of MRI evaluations of 126 consecutive fatty masses. *American Journal of Roentgenology*.

[7] Anderson S, Schwab C, Stauffer E, Banic A, Steinbach L (2001). Hibernoma: imaging characteristics of a rare benign soft tissue tumor. *Skeletal Radiology*.

[8] Munk PL, Lee MJ, Janzen DL (1997). Lipoma and liposarcoma: evaluation using CT and MR imaging. *American Journal of Roentgenology*.

[9] Vortmeyer AO, Böni R, Pak E, Pack S, Zhuang Z (1998). Multiple endocrine neoplasia 1 gene alterations in MEN1-associated and sporadic lipomas. *Journal of the National Cancer Institute*.

[10] Marini F, Falchetti A, Luzi E (2008). *Multiple Endocrine Neoplasia Type 1 (MEN1) Syndrome*.

[11] Darling TN, Skarulis MC, Steinberg SM, Marx SJ, Spiegel AM, Turner M (1997). Multiple facial angiofibromas and collagenomas in patients with multiple endocrine neoplasia type 1. *Archives of Dermatology*.

[12] Mertens F, Rydholm A, Brosjo O, Willen H, Mitelman F, Mandahl N (1994). Hibernomas are characterized by rearrangements of chromosome bands 11q13-21. *International Journal of Cancer*.

[13] Nord KH, Magnusson L, Isaksson M (2010). Concomitant deletions of tumor suppressor genes *MEN1* and *AIP* are essential for the pathogenesis of the brown fat tumor hibernoma. *Proceedings of the National Academy of Sciences of the United States of America*.

[14] Dreijerink KMA, Varier RA, van Beekum O (2009). The multiple endocrine neoplasia type 1 (MEN1) tumor suppressor regulates peroxisome proliferator-activated receptor *γ*-dependent adipocyte differentiation. *Molecular and Cellular Biology*.

[15] Gisselsson D, Höglund M, Mertens F, Dal Cin P, Mandahl N (1999). Hibernomas are characterized by homozygous deletions in the multiple endocrine neoplasia type I region: metaphase fluorescence in situ *Hybridization* reveals complex rearrangements not detected by conventional cytogenetics. *The American Journal of Pathology*.

